# Systematic analysis of volumetric ultrasound parameters for markerless 4D motion tracking

**DOI:** 10.1007/s11548-022-02665-5

**Published:** 2022-05-21

**Authors:** Johanna Sprenger, Marcel Bengs, Stefan Gerlach, Maximilian Neidhardt, Alexander Schlaefer

**Affiliations:** Institute of Medical Technology and Intelligent Systems, Hamburg University of Technology, Hamburg, Germany

**Keywords:** Ultrasound, Tracking, Motion estimation, Radiotherapy, Image guidance

## Abstract

**Objectives:**

Motion compensation is an interesting approach to improve treatments of moving structures. For example, target motion can substantially affect dose delivery in radiation therapy, where methods to detect and mitigate the motion are widely used. Recent advances in fast, volumetric ultrasound have rekindled the interest in ultrasound for motion tracking. We present a setup to evaluate ultrasound based motion tracking and we study the effect of imaging rate and motion artifacts on its performance.

**Methods:**

We describe an experimental setup to acquire markerless 4D ultrasound data with precise ground truth from a robot and evaluate different real-world trajectories and system settings toward accurate motion estimation. We analyze motion artifacts in continuously acquired data by comparing to data recorded in a step-and-shoot fashion. Furthermore, we investigate the trade-off between the imaging frequency and resolution.

**Results:**

The mean tracking errors show that continuously acquired data leads to similar results as data acquired in a step-and-shoot fashion. We report mean tracking errors up to 2.01 mm and 1.36 mm on the continuous data for the lower and higher resolution, respectively, while step-and-shoot data leads to mean tracking errors of 2.52 mm and 0.98 mm.

**Conclusions:**

We perform a quantitative analysis of different system settings for motion tracking with 4D ultrasound. We can show that precise tracking is feasible and additional motion in continuously acquired data does not impair the tracking. Moreover, the analysis of the frequency resolution trade-off shows that a high imaging resolution is beneficial in ultrasound tracking.

## Introduction

Ultrasound (US) offers non-invasive and non-ionizing imaging in real-time. These advantages make US one of the most frequently applied imaging modalities in various medical diagnostic tasks. Moreover, US is also frequently used for image guidance. While 2D US has been considered for different applications, recent advances make fast volumetric (4D) US interesting for motion tracking. Precise motion tracking is especially important when target movements may affect the quality of the treatment. One particular application is radiation therapy, where motion can severely affect the dose delivered to a target and cause severe side effects to surrounding healthy tissue. Approaches to mitigate the impact of motion are therefore widely used when delivering stereotactic body radiation therapy (SBRT). Active motion compensation requires knowledge of the internal target motion throughout a treatment fraction. Approaches based on X-ray imaging correlated with external motion surrogates from cameras are now widely used to monitor respiratory motion in clinics [1]. However, X-ray imaging requires the use of fiducial markers, particularly in the abdomen, and the infrequent imaging can lead to correlation errors.

Integrating MRI and linacs has also been considered for monitoring the 3D organ motion during treatment [2]. However, these systems are still complex and expensive. US motion tracking can be integrated more easily into existing setups [3–5] and allows for direct motion estimation of the target. This requires reproducible probe positioning and contact between the US probe and the patient. Seitz et al. propose for example a robot-based breathing and motion control and apply low contact forces while changing the probe positioning [6]. Previous work considers model probes for reproducible tissue deformations during treatment planning and delivery [7, 8]. Also, the ultrasound robot’s pose needs to be considered with respect to treatment plan quality [9]. Volumetric US has been considered for target tracking during radiotherapy [10, 11] and previous studies evaluate systems and methods for motion estimation in 3D or 4D US [12–14]. Ipsen et al. [15] compared for example different 4D US systems regarding their suitability in radiotherapy by assessing volume size, frame rates and image quality. Bell et al. [16] investigated different volume sampling rates for tracking in the context of respiratory motion, showing that sampling rates of 4 Hz to 12 Hz are required.

While US has advantages and in principle allows for markerless motion tracking, the evaluation of the tracking accuracy remains difficult, particularly as many studies rely on manual or indirect annotations with limited accuracy [14]. This complicates a systematic quantitative analysis, for example, to investigate to what extent motion artifacts or image quality impact the tracking performance. We perform a quantitative analysis of markerless volumetric US tracking and study the impact of different system parameters. First, we describe an experimental setup to automatically acquire 4D US data with accurate ground truth motion. Second, we investigate the influence of motion artifacts in continuously acquired US images by comparing the images to data acquired in a step-and-shoot fashion. Third, we vary the number of beams during imaging to assess the trade-off between imaging speed and resolution. Our analysis is based on well-established filters and considers real-world motion traces recorded during radiation therapy.

## Material and methods

### Experimental setup

**Fig. 1:**
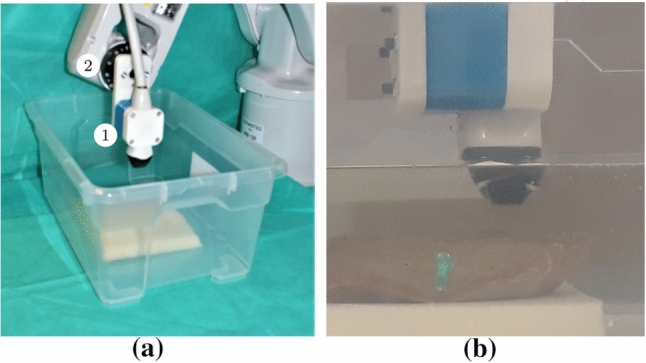
Our experimental setup (**a**). The US probe (1) is mounted to the robot (2) and positioned above the water tank. The liver is fixated to foam to prevent it from moving or floating (**b**)

Our experimental setup is based on an US system (Griffin, Cephasonics Ultrasound) and a robot arm (IRB 120, ABB) with a high repeatability of 0.01 mm. A matrix transducer (custom volume probe, Vermon) with a center frequency of 3 MHz is mounted to the end-effector of the robot with a 3D printed probe holder as shown in Fig. 1. The US probe is aligned to the robots’ axes and a plastic tank is placed beneath the probe which contains a foam layer. During our experiments we fixate the different phantoms with needles to the foam layer to prevent them from moving or floating. Subsequently, the plastic tank is filled with water to enable contactless US imaging of our phantoms. We apply a homogeneous speed of sound of 1540 ms$^{-1}$, as suitable for the tissue samples.

**Fig. 2:**
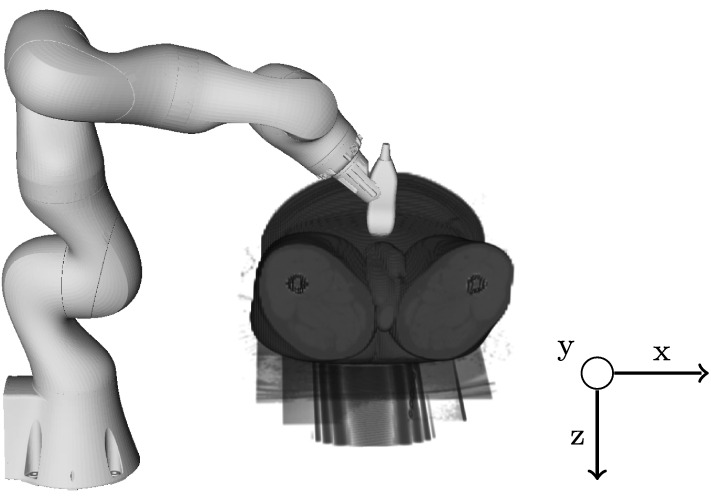
General setup for US tracking during radiotherapy. The US probe is connected to a robot and placed on the patient for contact during image acquisition

**Fig. 3:**
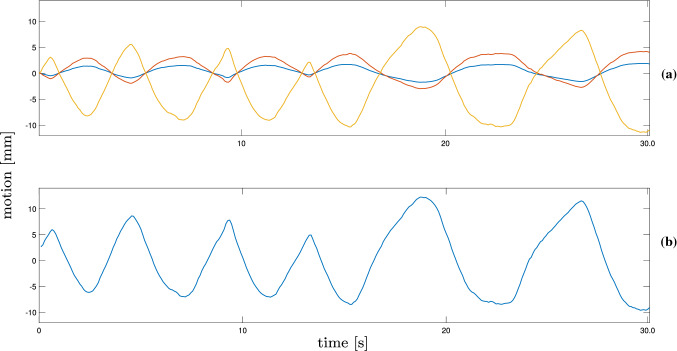
Exemplary trajectory for patient liver motion during free breathing in radiotherapy. The motion for x (blue), y (yellow) and z (red) is shown (**a**) as well as the main motion component of the three dimensions after applying a PCA (**b**)

**Table 1:** Mean and standard deviation of the amplitude, as well as minimum and maximum amplitude from the main motion component are reported in mm as well as the number of breathing cycles and the duration of the measurements in seconds of the trajectories for the markerless liver measurements

Trajectory	Phantom	Cycles	Mean	Std	Min	Max	Duration
1	Liver & marker	3.5	6.96	0.19	6.47	7.13	18.1
2	Liver	3	14.00	1.41	12.13	15.58	13.7
3	Liver & marker	4	13.50	0.50	13.26	14.64	17.8
4	Liver	4	9.53	0.34	9.25	10.14	17.3
5	Liver	4	10.73	3.32	7.72	16.11	18.4
6	Liver	2	17.68	1.01	16.66	18.26	15.2
7	Liver & marker	3	10.20	3.00	6.98	13.56	19.0
8	Liver & marker	3.5	14.45	2.32	11.88	15.58	15.7

### Motion traces

We consider US tracking in radiation therapy and use real three-dimensional motion traces that were acquired during treatment with a CyberKnife system at Georgetown University Hospital [17]. Figure 3a visualizes the different magnitudes in an exemplary trajectory. The motion is largest in US y-direction (superior-inferior), medium in z-direction (anterior-posterior) and smallest in x-direction (medial-lateral) in the available trajectories. For a more intuitive visualization and comparison of the tracking results to the ground truth position, we apply a principal component analysis (PCA) to determine the main motion component of the trajectories [17]. Note, that we do not apply the PCA to the US data and that the main motion component rather underestimates the motion magnitude. An example is given in Fig. 3b, the motion mainly reflects the superior-inferior motion from the patients. Figure 2 shows the general setup of US tracking during radiotherapy, the coordinate system indicates the US probe axes relative to a patient’s orientation. The motion traces were acquired from the liver of different patients during free breathing. We record data from eight different trajectories with bovine liver and four trajectories with a spherical marker. Table 1 reports the trajectories along with their mean and standard deviation of the amplitude, the minimum and maximum amplitudes as well as the durations of the recordings. The values are reported based on the main motion component after applying the PCA. The trajectories were selected to show different behavior concerning the trajectory course and maximum amplitude.

### Data acquisition

We use SUPRA [18] for 3D US imaging with beamforming, resulting in volumes of $268 \times 268 \times 268$ voxels covering a field-of-view (FOV) of $40 \times 40 \times 40$mm$^{3}$. Prior to each experiment, we manually position the robot about 10 mm above the phantom surface to ensure it is visible in the US FOV throughout the measurement. We record data from different tissue samples and different tissue regions. The recordings for each trajectory start at the same region-of-interest (ROI) from one tissue sample. After obtaining all measurements from one trajectory, another ROI was selected for subsequent measurements to evaluate ROIs with different tissue features. During data acquisition, the robot moves the US transducer along predefined trajectories while US images are acquired. The robot positions serve as ground truth for tracking. We systematically record and evaluate different system settings. First, we record data from markerless bovine liver tissue and from a spherical marker with a diameter of 2 mm.

Second, we acquire US data continuously and in a step-and-shoot fashion to investigate to what extent motion artifacts influence the tracking. Considering the data acquisition in a step-and-shoot fashion, we move the robot to a position along the trajectory, acquire an US volume and log the position before moving the robot to the next position. When acquiring data continuously, we move the robot in real-time along the trajectories while continuously recording US volumes and logging the robot positions, as well as the timestamps from both systems. The positions are matched to the US volumes based on the timestamps. For comparison, we sample the trajectory points for the step-and-shoot measurements with the corresponding imaging frequencies.

**Fig. 4:**
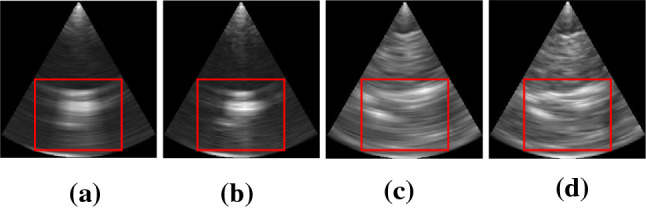
Slices from US volumes showing the spherical marker (**a** and **b**) and exemplary bovine tissue (**c** and **d**) with 8 $\times$ 8 beams and 16 $\times$ 16 beams, respectively. The red boxes indicate the crop used for tracking

Third, we vary the number of beams used for US imaging between $16 \times 16$ and $8 \times 8$ beams to investigate the trade-off between imaging speed and resolution. A lower imaging frequency but higher resolution is obtained with $16 \times 16$ beams, while $8 \times 8$ beams lead to a higher imaging frequency but lower resolution, generally showing less details. The maximum amount of data and the imaging frequency were limited by the system buffer. While using $8 \times 8$ beams enables continuous imaging of up to 44 Hz, the frequency was limited to 22 Hz for storing acquired data. Furthermore, imaging with $8 \times 8$ beams limited the continuous data acquisition to at most 20 s due to the system buffer. Figure 4 shows examples for US images taken of the marker with $8 \times 8$ beams and $16 \times 16$ beams, respectively, and corresponding examples of bovine liver. The slices are extracted from the center of the volume. The signal in the images of the bovine liver is based on the markerless structure of the tissue without observing specific landmarks.

### Methods for motion estimation

We apply two different methods for motion estimation using the 3D and 4D aspect of the data. Methods based on normalized cross-correlation (NCC) have previously been applied for US tracking [19, 20]. The motion between the first volume (template) is compared to every succeeding volume along the trajectory. Furthermore, a MOSSE filter [21] is applied to the US data which takes into account the temporal dimension of the data. We apply a preprocessing to reduce speckle noise. For this purpose, we use a median filter of kernel size $3 \times 3 \times 3$ and a window leveling for contrast enhancement. Furthermore, we crop the data to cuboids of $158 \times 158 \times 118$ voxels, as indicated by the red boxes in Fig. 4, to avoid the presence of the edges from the conical appearance of the US data in the volume.

#### Normalized cross-correlation

We implement normalized cross-correlation via

1 $$\begin{aligned} r(x,y,z) = {\mathcal {F}}^{-1}({\mathcal {F}}(f(x,y,z)) \circ {\mathcal {F}}(v(x,y,z))^{*}) \end{aligned}$$

with ${\mathcal {F}}$, the Fourier transform, *f*(*x*, *y*, *z*) and *v*(*x*, *y*, *z*) as the template and reference volume and $\circ$, the element wise multiplication. The translation between the volumes is indicated by a peak at the corresponding position in *r*.

**Table 2:** Tracking error $e_{t}$ in mm for evaluation of the different US data with MOSSE and NCC for the spherical marker. Continuously acquired data is compared to data acquired in a step-and-shoot fashion, as well as the different number of beams used for imaging

Traj.	Acquisition	NCC $8\times 8$	MOSSE $8\times 8$	NCC $16\times 16$	MOSSE $16\times 16$
1	Step & shoot	0.65 ± 0.30	0.66 ± 0.46	0.25 ± 0.08	0.21 ± 0.08
	Continuous	0.67 ± 0.77	0.81 ± 0.93	0.84 ± 0.60	0.60 ± 0.22
3	Step & shoot	0.58 ± 0.33	1.20 ± 0.84	0.28 ± 0.19	0.55 ± 0.69
	Continuous	0.75 ± 0.24	1.16 ± 3.39	0.32 ± 0.25	0.44 ± 0.38
7	Step & shoot	0.76 ± 0.25	1.16 ± 0.49	0.27 ± 0.10	0.45 ± 0.18
	Continuous	0.60 ± 0.38	0.91 ± 0.58	0.67 ± 0.53	0.79 ± 0.50
8	Step & shoot	0.67 ± 0.35	1.34 ± 0.67	0.33 ± 0.19	0.66 ± 0.48
	Continuous	0.85 ± 1.21	1.52 ± 1.53	0.58 ± 0.56	0.82 ± 0.60
Mean	Step & shoot	0.66 ± 0.31	1.09 ± 0.61	0.28 ± 0.14	0.47 ± 0.36
	Continuous	0.72 ± 0.65	1.10 ± 1.61	0.60 ± 0.48	0.66 ± 0.43

#### MOSSE

We implement the MOSSE filter with 3D operations based on Bolme et al. [21]. Initially, the MOSSE filter needs to be trained on example images $f_{i}$ and training outputs $g_{i}$. We use a 3D Gaussian peak at the center of the shifted ROI. The filter *H* is defined as

2 $$\begin{aligned} H^{*}_{i} = \frac{G_{i}}{F_{i}} \end{aligned}$$

and maps the training images to their outputs. $F_{i}$ and $G_{i}$ are the Fourier transform of $f_{i}$ and $g_{i}$. During tracking, the filter can be adapted to the input to account for variation in the target appearance. With learning rate $\eta$ the filter is updated as

3 $$\begin{aligned} H_{i}^{*} = \frac{A_{i}}{B_{i}} \end{aligned}$$

with $A_{i} = \eta \, G_{i} \circ F_{i}^{*} + (1 - \eta ) \, A_{i-1}$ and $B_{i} = \eta \, F_{i} \circ F_{i}^{*} + (1 - \eta ) \, B_{i-1}$. Motion shifts can then be detected by computing

4 $$\begin{aligned} r(x,y,z) = {\mathcal {F}}^{-1}({\mathcal {F}}(f(x,y,z)) \circ {\mathcal {F}}(h(x,y,z))^{*}) \end{aligned}$$

for new input images.

#### Evaluation

The difference in position between two US volumes can be determined using the corresponding robot positions. Following the same evaluation as in [14], the error between ground truth and estimation is calculated as

5 $$\begin{aligned} e_{t} = \Vert p_{t} - {\hat{p}}_{t} \Vert . \end{aligned}$$

$e_{t}$ is the Euclidean norm of the difference between the real motion shift $p_{t}$ and the predicted motion shift ${\hat{p}}_{t}$ at time *t*.

**Fig. 5:**
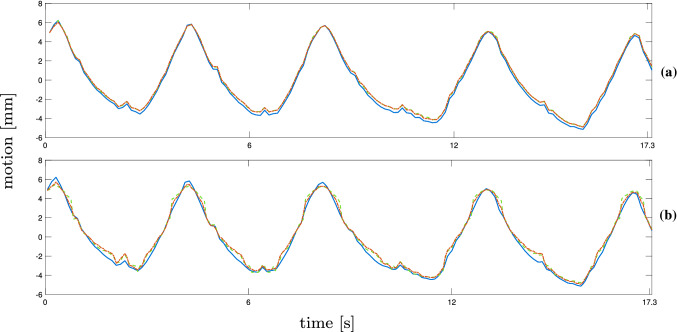
Results for NCC (red, dotted) and MOSSE (green, dotted) for step-and-shoot data set with the ground truth (blue). The main motion component of the trajectories and tracking results are shown for trajectory 4 for $16 \times 16$ beams (**a**) and $8\times 8$ beams (**b**)

**Fig. 6:**
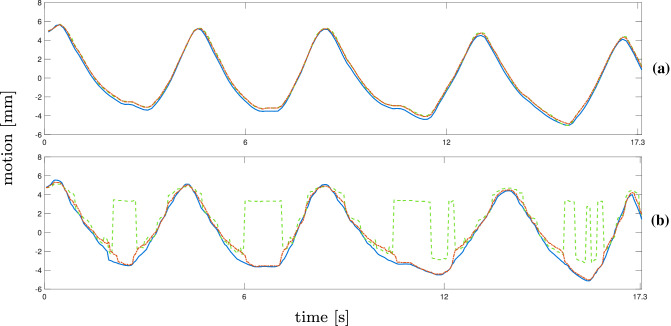
Results for NCC (red, dotted) and MOSSE (green, dotted) for continuous data with the ground truth (blue). The main motion component of the trajectories and tracking results is shown for trajectory 4 for $16 \times 16$ beams (**a**) and $8\times 8$ beams (**b**)

## Results

Initially, we evaluate the data acquired from the spherical marker and investigate to what extent motion tracking is feasible with the methods. The results for the tracking errors are presented in Table 2. NCC and MOSSE are used to evaluate data acquired with $8 \times 8$ beams and $16 \times 16$ beams in a step-and-shoot fashion or continuously for four different trajectories. Considering the mean Euclidean error of the four measurements, we obtain 0.72 mm and 1.10 mm for NCC and MOSSE, respectively, for $8 \times 8$ beams on the continuous data. The results are similar to the errors obtained for the step-and-shoot recordings, 0.66 mm and 1.09 mm. The evaluation on the data acquired with $16 \times 16$ beams leads to lower tracking errors, especially for step-and-shoot recordings. We obtain tracking errors of 0.28 mm and 0.47 mm as well as 0.60 mm and 0.66 mm for step-and-shoot and continuous recordings, respectively. The errors for the individual trajectories differ from the mean errors but are in general low with regard to the mean amplitude values reported in Table 1.

Subsequently, we evaluate the tracking results for markerless liver tissue. The overall mean tracking errors are increased compared to the marker-based tracking results. For $8 \times 8$ beams, we report tracking errors of 2.60 mm and 2.52 mm as well as 2.01 mm and 2.15 mm for step-and-shoot and continuous data, respectively. The errors for the continuous data are slightly lower. Considering $16 \times 16$ beams, we obtain errors of 1.85 mm and 0.98 mm for step-and-shoot data and 1.82 mm and 1.36 mm for continuous data. The data acquired with higher resolution, $16 \times 16$ beams, lead to more precise tracking results than the lower resolution, $8 \times 8$ beams. The tracking errors for the individual trajectories differ strongly from the mean error values. Furthermore, we observe outliers in the tracking results for individual trajectories. The results obtained for trajectory 6 show for example a wider value range and outliers for the NCC step-and-shoot results.

**Table 3:** Tracking error $e_{t}$ in mm for evaluation of the different US data with MOSSE and NCC for markerless liver tissue. Continuously acquired data is compared to data acquired in a step-and-shoot fashion, as well as the different number of beams used for imaging

Traj.	Acquisition	NCC $8\times 8$	MOSSE $8\times 8$	NCC $16\times 16$	MOSSE $16\times 16$
1	Step & shoot	0.28 ± 0.12	0.56 ± 0.27	0.21 ± 0.06	0.27 ± 0.09
	Continuous	0.52 ± 0.19	0.53 ± 0.27	0.21 ± 0.10	0.45 ± 0.29
2	Step & shoot	3.75 ± 2.51	3.28 ± 3.08	2.29 ± 2.57	0.67 ± 0.98
	Continuous	3.10 ± 2.58	1.55 ± 1.35	0.80 ± 0.62	0.74 ± 0.84
3	Step & shoot	3.05 ± 3.50	3.59 ± 4.25	2.63 ± 3.51	2.73 ± 4.03
	Continuous	2.78 ± 3.36	3.52 ± 3.75	2.83 ± 3.50	3.65 ± 4.07
4	Step & shoot	0.70 ± 0.24	0.93 ± 0.67	0.32 ± 0.10	0.36 ± 0.11
	Continuous	0.62 ± 0.41	2.12 ± 2.51	0.25 ± 0.07	0.30 ± 0.10
5	Step & shoot	2.56 ± 1.81	3.34 ± 2.35	1.11 ± 1.48	0.68 ± 0.92
	Continuous	2.04 ± 1.31	2.24 ± 1.27	1.78 ± 1.83	0.82 ± 0.71
6	Step & shoot	4.60 ± 2.96	2.29 ± 2.37	4.02 ± 3.71	1.43 ± 2.40
	Continuous	1.59 ± 1.82	1.59 ± 1.51	2.45 ± 3.01	0.78 ± 0.95
7	Step & shoot	1.13 ± 0.67	1.49 ± 0.95	0.56 ± 0.28	0.65 ± 0.36
	Continuous	1.09 ± 0.69	1.25 ± 0.89	0.50 ± 0.26	0.61 ± 0.36
8	Step & shoot	4.73 ± 3.13	4.71 ± 3.43	3.69 ± 3.02	1.07 ± 2.36
	Continuous	4.35 ± 4.24	4.39 ± 3.77	5.78 ± 4.67	3.56 ± 4.21
Mean	Step & shoot	2.60 ± 1.87	2.52 ± 2.17	1.85 ± 1.84	0.98 ± 1.41
	Continuous	2.01 ± 1.83	2.15 ± 1.91	1.82 ± 1.76	1.36 ± 1.44

**Fig. 7:**
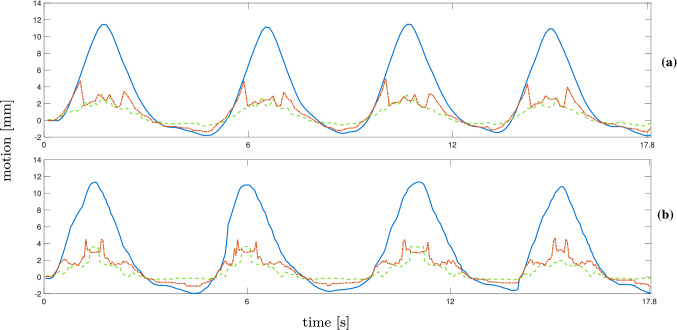
Results for NCC (red, dotted) and MOSSE (green, dotted) for continuous data with the ground truth (blue). The main motion component of the trajectories and tracking results are shown for trajectory 3 for $16 \times 16$ beams (**a**) and $8\times 8$ beams (**b**)

Examples for the resulting trajectories from markerless tracking with bovine liver are shown in Figs. 5 and 6 . The main motion component, after applying a PCA to the tracking estimates, is visualized. Figure 5a and b displays the main motion component of trajectory number 4 and the tracking results for NCC and MOSSE for both resolutions (step-and-shoot). The motion is determined precisely with both methods except for a few minor discrepancies. The results for $8 \times 8$ beams in Fig. 5b show a few more inaccuracies and higher deviation from the ground truth. In Fig. 6a and b, estimations from continuous data for trajectory 4 are displayed. The MOSSE filter is able to estimate motion from higher-resolution images, but shows failures at the peaks of the trajectory when applied to the lower resolution data. This is reflected in the tracking error for trajectory 4 in Table 3. The estimated trajectory shows failures at every peak, leading to a square-like course of the tracking estimate. An example for unsuccessful tracking is given in Fig. 7a and b from trajectory number 3 for continuous data. The tracking results for MOSSE and NCC follow the ground truth motion only for small shifts but the steeper parts and peaks are not detected. Furthermore, both resulting trajectories show oscillations where the peaks could not be detected.

## Discussion

The results show that motion tracking is possible with the acquired 4D US tracking data set. The methods enable precise motion tracking for the marker data set but slight differences in the mean tracking errors between the data acquisition modes can be observed. The results differ strongly for the measurements in the markerless tissue and indicate that continuous data acquisition leads to slightly better results when using $8 \times 8$ beams. For $16 \times 16$ beams, continuous data leads to similar or slightly worse results. The additional motion in the continuous data does not affect the tracking precision. A higher resolution seems beneficial for more precise tracking results. Bell et al. [16] indicated that imaging frequencies between 8 and 12 Hz are required for tracking breathing motion. Our results confirm that an imaging rate of 11 Hz is sufficient. Considering a clinical setting, system latencies can lead to higher errors in general. This is not reflected in our experiments and using a higher frequency could be beneficial in such scenarios.

In comparison with the results from the 2015 MICCAI CLUST workshop [14], our best mean tracking error is substantially lower. The mean tracking errors are reported for two approaches with 1.74 mm and 1.80 mm. However, note that the acquisition of US data is different regarding, for example, the size of the FOV and image resolution. While our ground truth is precisely generated with a robot and does not suffer from subjective evaluation or inter-observer variability, De Luca et al. also report the mean Euclidean errors of three observers in the range of 1.19 mm to 1.36 mm. In our experiments the probe is static. However, considering a clinical setting, contact forces between probe and patient can be measured to move the robot and follow the patient’s motion. Tissue deformations can occur close to the probe position which is not relevant when considering the crop we use for tracking.

Trajectory 3 and 8 lead to especially high tracking errors for the different settings. The visualizations from trajectory 3 show difficulties at the peaks and the mean values reported in Table 1 along with the number of cycles and the maximum indicate a steep course of the trajectories. One possible source of error is the smaller overlap between the template and following volumes when the motion is larger. The results obtained for the marker show that the different trajectories can generally lead to precise tracking results, but estimating motion based on markerless tissue structures is more challenging. The tissue appearance from different ROIs can cause failures when the features are not suitable for the applied method. Furthermore, we compare the initial US template against the subsequent US volumes for tracking. In case of a noisy template volume, the tracking could therefore be impaired for the whole trajectory. The difference between the two methods is visible in Fig. 6b. NCC is less precise for $8 \times 8$ beams, but MOSSE fails to predict the peaks and the general course of the estimated trajectory is noisier. This could be due to non-optimal filter adaptions over time when motion is not detected.

The influence of the ROI and the initial template needs to be investigated further. Additional filtering of the tracking estimates or outlier rejection schemes can help to improve the precision and robustness of the tracking approaches. Since we do not aim to implement precise tracking but want to analyze the different system settings based on the tracking results, we do not apply methods for outlier rejection in this work. Future work could consider convolutional neural networks (CNNs) for precise tracking. Previous work in ultrasound tracking [22, 23] has shown the potential of CNNs in ultrasound tracking and our setup is suitable to automatically acquire large data sets for training CNNs. Our experimental setup allows following motion for longer time periods in general. Since we perform a quantitative analysis we record the data for an offline analysis which limits the possible acquisition duration due to the system buffer.

## Conclusion

We perform a quantitative analysis of markerless volumetric US tracking for radiotherapy. We compare different imaging resolutions and evaluate the influence of motion artifacts. The results show that a high imaging resolution is advantageous compared to a higher imaging rate considering the present motion traces from radiotherapy treatment. The continuously acquired data lead to similar tracking errors and enable tracking of markerless tissue. In general, the tracking performance is reduced for certain trajectories. In the future, the setup can be used to further analyze system parameters for US tracking. Data from different trajectories can be recorded and failures during tracking investigated thoroughly to improve the methodical development for US tracking in radiotherapy.
